# Anisotropic Porosity and Interface Synergy Enhanced Gas Permselectivity in Heterolayer Metal‐Organic Framework Membrane

**DOI:** 10.1002/chem.202403607

**Published:** 2024-11-12

**Authors:** Susmita Kundu, Tanmoy Maity, Suvendu Panda, Ritesh Haldar

**Affiliations:** ^1^ Tata Institute of Fundamental Research Hyderabad Gopanpally Hyderabad 500046 Telangana India

**Keywords:** MOF heterostructure, UiO-66, ZIF-8-NH_2_, Permselectivity, Anisotropic porosity

## Abstract

The pursuit of sustainable, carbon‐free separation technology hinges on the efficient separation of gas mixtures with high separation factors and flow rates, i. e. high permselectivity. However, achieving this objective is arduous due to the meticulous engineering at the angstrom scale and intricate chemical manipulation required to design the pores within membranes. To address this challenge, a proof‐of‐concept for an anisotropic porous membrane has been devised. Employing a meticulous step‐by‐step methodology, two distinct porous metal‐organic frameworks (MOFs) are integrated to form a monolithic anisotropic membrane. By harnessing pore anisotropy (3.4 to 6 Å) aligned with the gas permeation direction and a unique interface characterized by cross‐linked pores derived from the two distinct MOFs, this membrane transcends the performance limitations inherent in the individual MOF membranes (~45 % enhanced selectivity). This approach not only sheds light on the heterolayer membrane design strategy but also elucidates the intricate CO_2_/N_2_ permselectivity relationship inherent in the interface structure.

## Graphical abstract

An anisotropic heterolayer metal‐organic framework (MOF) membrane, composed of UiO‐66‐NH_2_ and ZIF‐8, is synthesized using a sequential growth strategy. The membrane's pore anisotropy (ranging from 3.4 to 6 Å) and a distinct MOF‐MOF interface with chemically crosslinked pores demonstrate enhanced CO_2_/N_2_ permselectivity compared to the performance of individual MOF membranes.

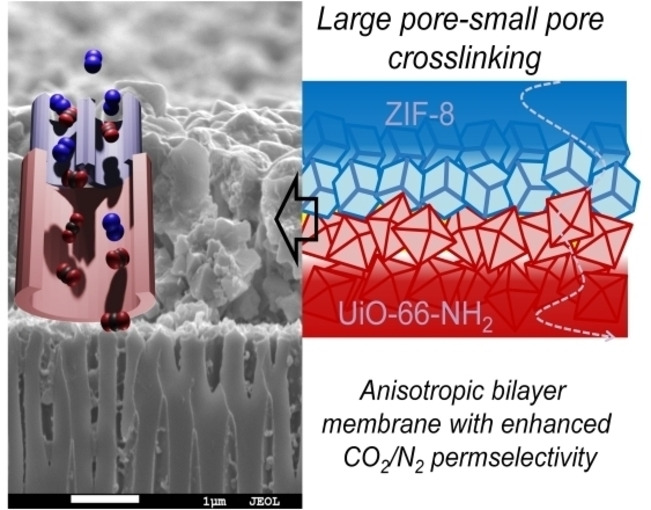

## Introduction

Efficiently achieving the separation of gases and liquids with minimal energy input and a diminished carbon footprint is imperative but poses significant hurdles. Present methodologies rely on principles such as chemisorption and boiling points, yet porous membrane‐based separation offers a compelling alternative.[^1^, ^2^, ^3^, ^4^, ^5^] Through meticulous adjustment of pore dimensions and surface chemistry, these membranes can attain exceptional separation efficiency.[^6^, ^7^] Metal‐organic frameworks (MOFs) emerge as particularly promising due to their permanent porosity and customizable traits.[^8^, ^9^, ^10^] Integrating MOFs into membranes, either independently or within polymer composites, facilitates precise molecular sieving.[^9^, ^11^, ^12^, ^13^, ^14^] Nonetheless, challenges persist, including striking the right balance between selectivity and permeability, as well as overcoming scalability limitations. Surmounting these obstacles is paramount for the advancement of membrane‐based separation technology.

**Scheme 1:**
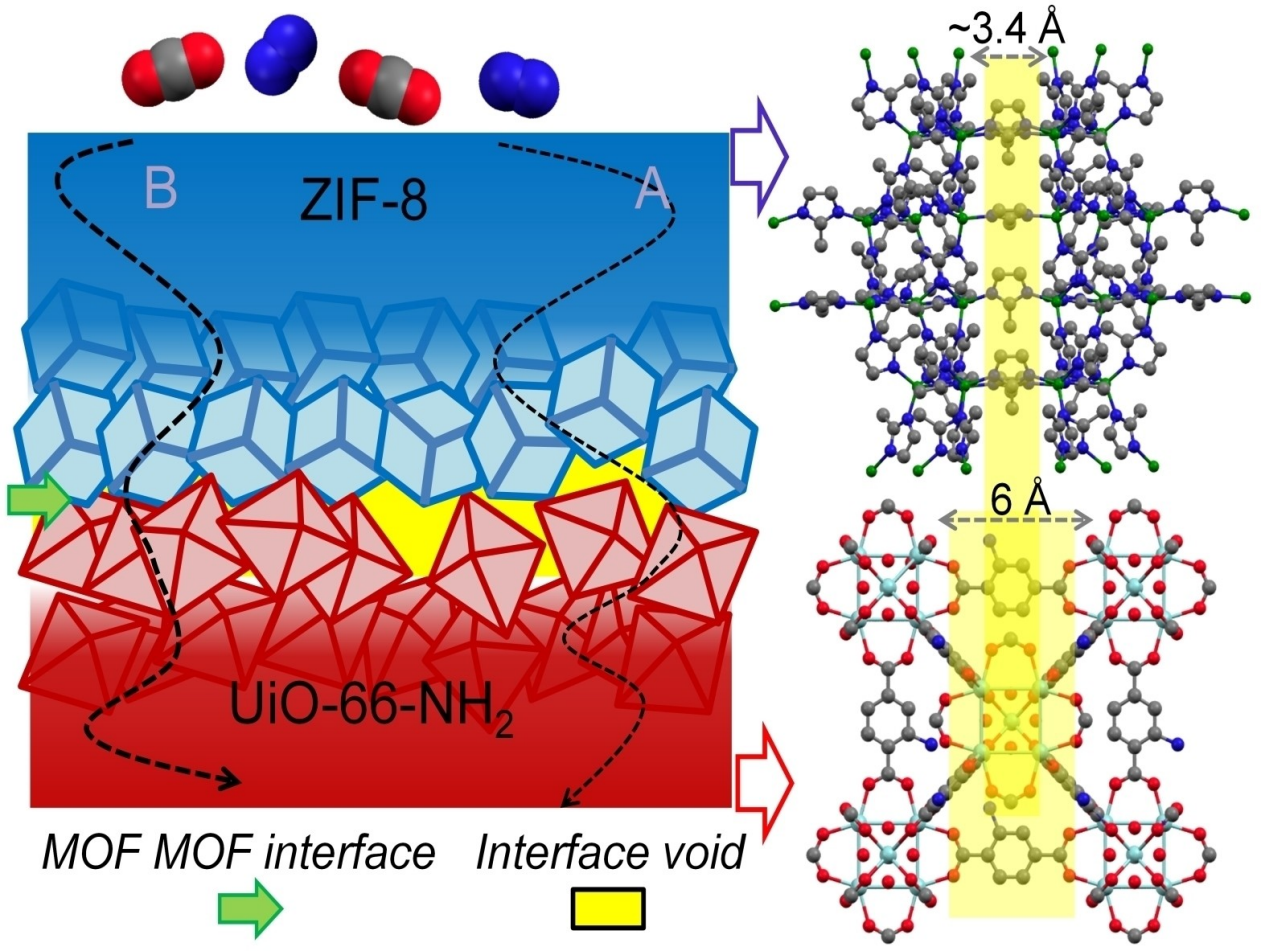
Schematic illustration of gas diffusion path in an anisotropic dual pore metal‐organic framework membrane made of ZIF‐8 and UiO‐66‐NH2; A and B are different diffusion path; right: yellow shaded region indicates dual porosity of the membrane. Zr=cyan, Zn=green, N=blue, C=gray, O=red in the ball and stick structure model.

To tackle the inherent compromise between selectivity and permeability, it is imperative to establish novel *de novo* principles for membrane design. Various strategies have already been identified for advanced applications of MOFs in devices such as transistors, diodes, and electrocatalysis.[^15^, ^16^, ^17^, ^18^, ^19^] These include multivariate design involving linker or metal node variations, post‐synthetic chemical adjustments, modulation of anisotropic morphology, and engineering defects.[^20^, ^21^, ^22^, ^23^, ^24^] These approaches regulate the local chemical structure of pores, introducing new interacting sites into the material. These strategies are also being explored for enhancing performance in MOF‐membrane design.[^25^, ^26^] In this communication, we delve into a subtler approach that circumvents direct chemical modification of the microstructure. We consider the integration of two distinctively porous MOFs as heterolayer structure with well‐defined interface. The concept of heterostructured MOFs has been extensively explored in core‐shell geometry, where a core MOF is enveloped by a shell MOF layer.[^27^, ^28^, ^29^, ^30^] In the context of permeation studies, diffusion occurs across two MOF‐MOF interfaces in core‐shell geometry, as seen in previously studied core‐shell MOF polymer composite membranes.[^31^, ^32^] However, in a heterolayer MOF‐on‐MOF structure,^[33]^ the permeation rate is influenced by individual layers with distinct porosity and functionality, as well as by a single interface (Scheme 1). In this investigation, we have designed a heterolayer MOF‐on‐MOF structure with anisotropic pore size distribution across its thickness. To examine the impact of this anisotropic pore geometry on gas permeation, we selected two robust and distinct MOF structures: ZIF‐8 (ZIF = zeolitic imidazolate framework) and UiO‐66‐NH_2_ (UiO=University of Oslo).[^34^, ^35^, ^36^] The pore window and cavity sizes are 3.4 and 12 Å for ZIF‐8, 6 and 11 Å, for UiO‐66‐NH_2_, respectively (also see Table S1). Our analysis of gas permselectivity with CO_2_ and N_2_ underscores the contrasting lattice dimensions, pore geometries, morphologies, and chemical functionalities inherent in these two MOFs. Through a step‐by‐step synthesis approach, we combined these MOFs to fabricate a heterolayer membrane. Our findings demonstrate that this anisotropic membrane effectively harnesses the favorable attributes of both MOFs, resulting in a significant improvement in CO_2_/N_2_ permselectivity, i. e., approximately 45 % selectivity enhancement, compared to the sieving MOF‐layer ZIF‐8. In the subsequent sections, we delineate our approach to designing the membrane and delve into the mechanisms through which pore anisotropy and interfacial interactions bolster the enhanced CO_2_/N_2_ permselectivity.

## Results and Discussion

### Anisotropic Porosity in Heterolayer Thin Film

For epitaxial MOF‐on‐MOF growth, lattice match is a necessity. However, MOF lattices being more elastic compared to inorganic materials,[^37^, ^38^, ^39^] lattice distortion at the interface can allow mismatched crystal growth. This leads to interfacial defects and also crystal facet specific anisotropic growth,[^30^, ^40^] which often play a critical role in determining the functionality (e. g. selective adsorption, charge transport, catalysis).[^39^, ^41^, ^42^] In the present study, we have selected a pair of MOFs, ZIF‐8 and UiO‐66‐NH_2_, having very dissimilar lattices (sodalite and face‐centered cubic topology, respectively) and chemical structures (Zn^2+^, 2‐methyl imidazole [2‐meIm]; Zr^4+^, 2‐aminoterephthalic acid [NH_2_‐bdc], respectively).[^34^, ^36^] In the absence of requisite lattice match, a stable interface structure for this pair of MOFs can form via multiple noncovalent and coordination linkages. This type of heterolayer interface can have following structures: A) the resulting heterolayer generates intercrystalline void space at the interface, forming a core‐void‐shell structure. This configuration will promote nonselective permeation, allowing molecules to pass through indiscriminately. However, the presence of dangling reactive sites at the interface introduces a selective interaction site, influencing the diffusion process in a targeted manner. B) An alternative interface structure can be generation of even smaller pores (than those of the individual MOFs), due to pore off‐set. In this case also selectivity will enhance, and permeance will decrease. Scheme 1 depicts both of these scenarios. In the subsequent discussions, we have considered both of these plausible interface structures and correlated with the experimental findings. Note that an earlier study on core‐shell type ZIF‐8‐UiO‐66‐NH_2_ indicated ~50 % enhanced CO_2_/N_2_ permselectivity is due to small pores generated at the MOF‐MOF interface.^[31]^

**Figure 1:**
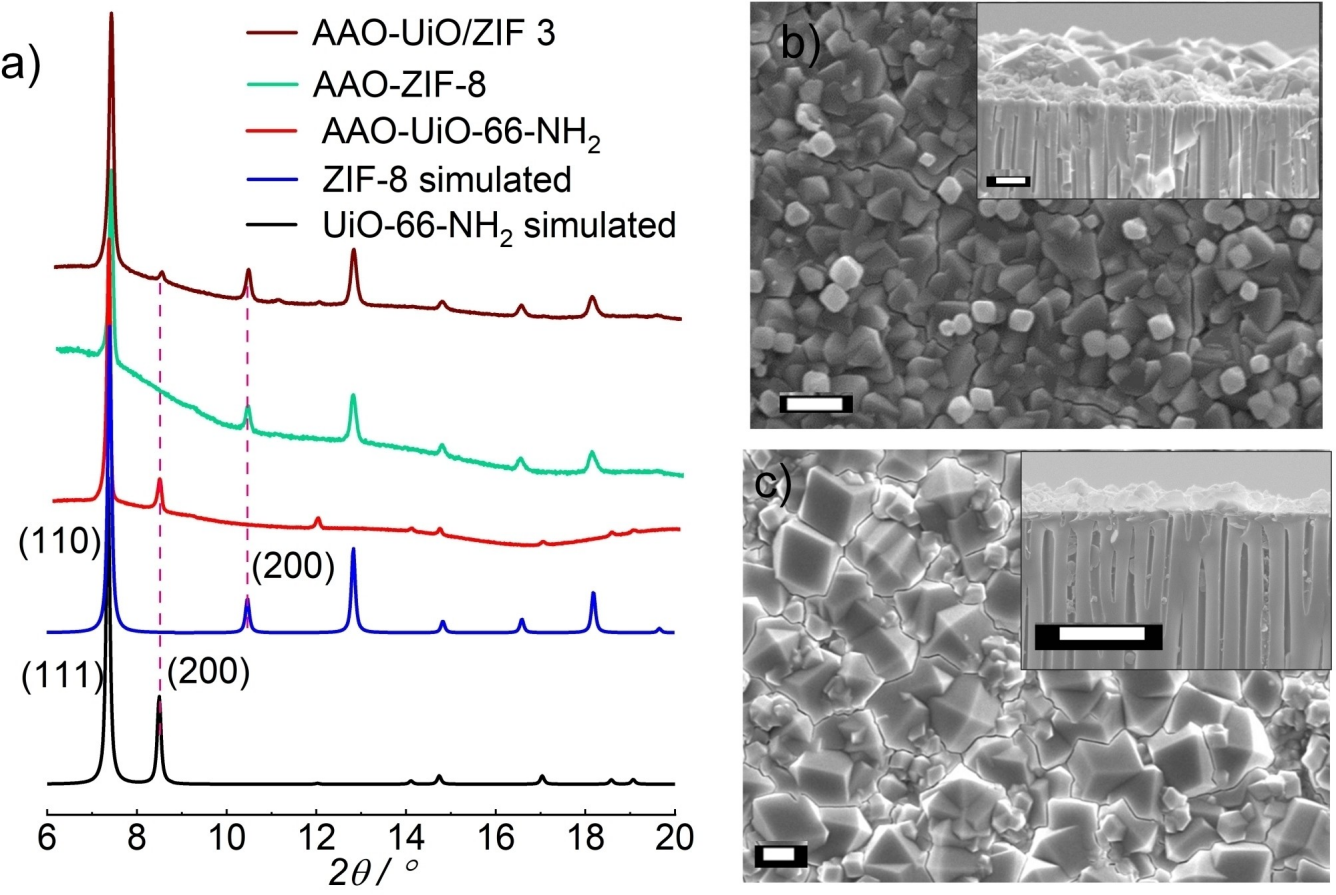
a) XRD patterns of ZIF‐8 and UiO‐66‐NH_2_ simulated, experimental and UiO/ZIF 3 grown on AAO, b‐c) SEM morphology and cross‐section (inset) of UiO‐66‐NH_2_ and ZIF‐8, respectively. Scale bar=1 μm.

**Figure 2:**
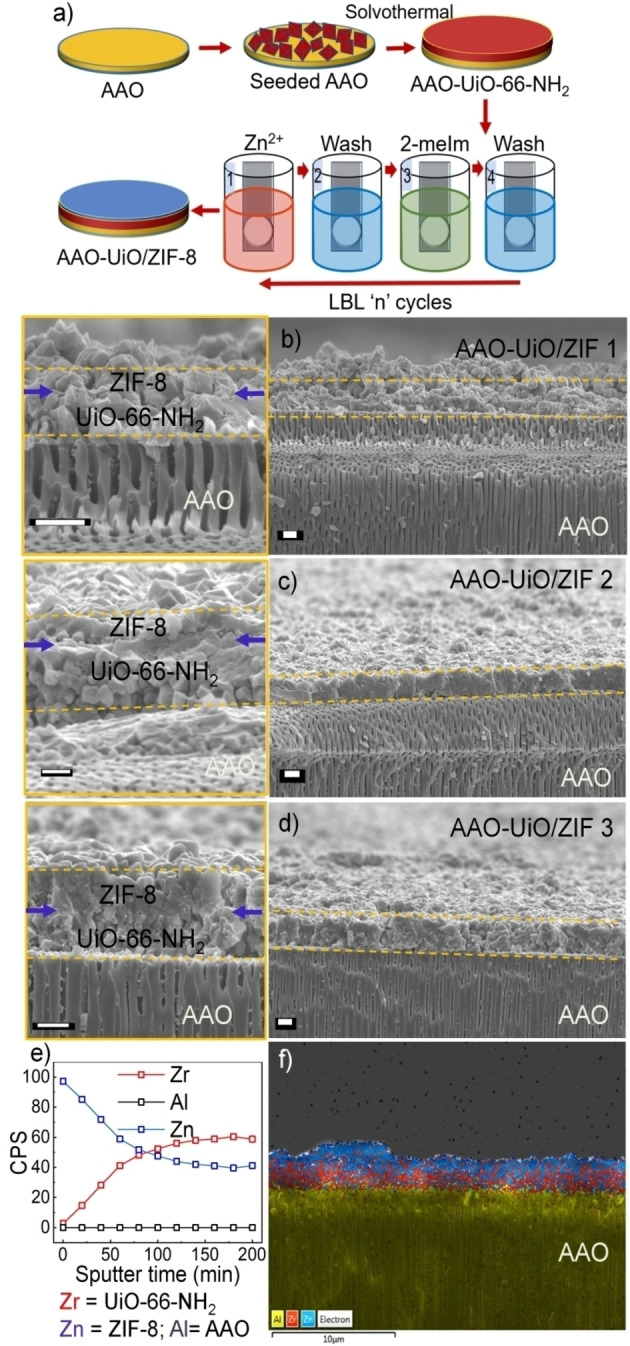
a) Schematic illustration of anisotropic membrane synthesis, LBL=layer‐by‐layer, b‐d) SEM cross‐sectional view of UiO/ZIF 1–3 membranes exhibiting distinct layers of ZIF‐8 and UiO‐66‐NH_2_ of varying thickness (left=enlarged image); yellow lines are indicative of the MOF membrane thickness. Scale bar=1 μm,e) Zn, Zr and Al signals in XPS during different sputtering time for UiO/ZIF 3, f) Zn, Zr and Al signals in EDX mapping for UiO/ZIF 3.

Initially, we have optimized the growth condition of the individual MOF layers on porous anodic alumina oxide (AAO; pore size ~200 nm). UiO‐66‐NH_2_ was grown as a continuous thin film on AAO using a seeded solvothermal methodology. In this method, the −OH functional AAO substrate was seeded with microcrystals of UiO‐66‐NH_2_ (see experimental section), followed by a solvothermal reaction at 120 °C. Without seeding, thin film deposition was found to be inhomogeneous (Figure S1). X‐ray diffraction (XRD) pattern of the AAO‐UiO‐66‐NH_2_ showed similar diffractions peaks as the simulated pattern, confirming the formation of phase pure MOF thin film (Figure 1a). Scanning electron microscopy (SEM) images of the membrane confirmed homogenous coverage and absence of any obvious pin holes (Figure 1b). To make continuous membrane of ZIF‐8 on −OH functionalized AAO substrate a layer‐by‐layer dip coating method was adopted.^[43]^ The synthesized AAO‐ZIF‐8 membrane was characterized by XRD and SEM. XRD pattern confirmed a ZIF‐8 crystalline phase (Figure 1a), and SEM images illustrated in Figure 1c confirmed highly intergrown pin hole free membrane. These optimized synthesis conditions were then used to grow the MOF‐on‐MOF membrane UiO‐66‐NH_2_ and ZIF‐8 (Figure 2a). Before the ZIF‐8 growth on the AAO‐UiO‐66‐NH_2_, membrane was thoroughly washed and solvent exchanged with methanol. This washing step is done to avoid any impure crystalline phase growth during the ZIF‐8 synthesis; i. e. mixing of Zn^2+^ with NH_2_‐bdc and Zr^4+^ with 2‐meIm. By employing different cycle growth of ZIF‐8, three different compositions (thicknesses) of the anisotropic membrane AAO‐UiO/ZIF 1–3 were synthesized (see experimental section, Figure S2–S3). The XRD pattern of the AAO‐UiO/ZIF 3, having thickest ZIF‐8 layer, indicated presence of crystalline phases of both of the individual MOFs with no preferential crystalline orientation (Figure 1a). The varying thicknesses of ZIF‐8 layers on the UiO‐66‐NH_2_ can be visualized from the cross‐section SEM images in Figure 2b–d. The SEM cross‐sectional and morphology image (Figure 2d and Figure S3) of AAO‐UiO/ZIF 3 membrane shows a monolithic, pinhole free form of the membrane. To check the anisotropy in the membrane structure, we have carried out a depth profile analysis of the AAO‐UiO/ZIF 3 membrane using x‐ray photoelectron spectroscopy (XPS). It clearly shows that with increasing ion sputtering time, Zn signal decays and Zr signal rises (Figure 2e). This confirmed the proposed MOF‐on‐MOF geometry. The elemental mapping (ZIF‐8 on top of UiO‐66‐NH_2_, grown on AAO) using energy dispersive x‐ray (EDX) also indicated a sharp Zr/Zn contrast confirming the distinct heterolayer membrane structure (Figure 2f). Note that in the absence of any lattice match of the MOF‐layers, a mixed‐layer interface is unlikely. A detail of the possible chemical structure of the interface will follow after the discussion on the gas permeation.

### Enhanced Gas Permselectivity

**Figure 3:**
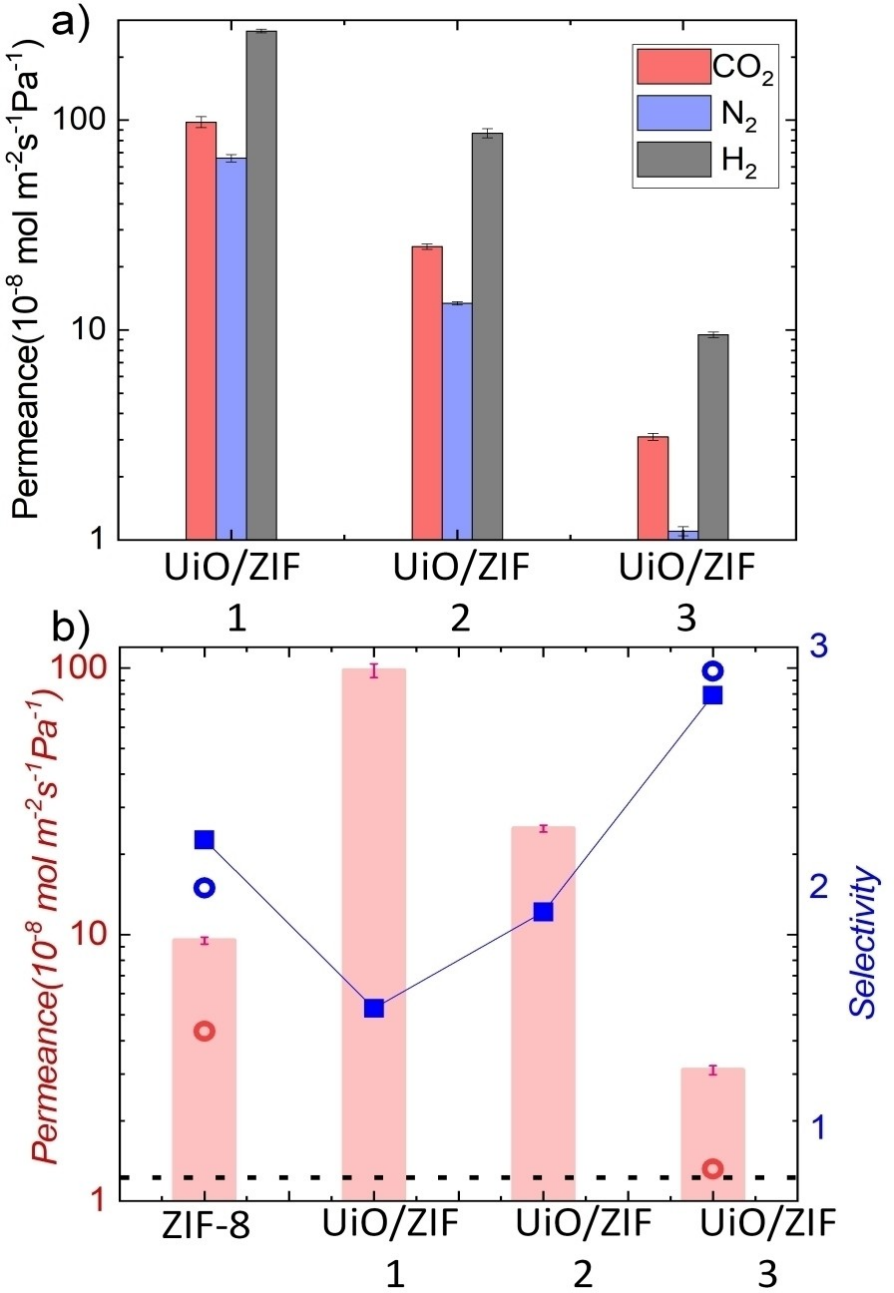
a) Single component H_2_, N_2_ and CO_2_ permeance of individual anisotropic MOF membranes (on AAO) at 303 K, b) CO_2_ permeance and CO_2_/N_2_ selectivity of the ZIF‐8 and anisotropic membranes at 303 K (single gas permeance=red bar, ideal selectivity=blue square), 50 : 50 CO_2_/N_2_ gas mixture CO_2_ permeance=red circle, and selectivity=blue circle. Dotted black line indicates CO_2_/N_2_ Knudsen selectivity.

Following the verification of anisotropic heterostructure formation, we proceeded to directly compare the gas permeation capabilities of the AAO‐UiO/ZIF 1–3 membranes with those of individual isotropic MOF membranes. We have selected H_2_ (2.89 Å), CO_2_ (3.3 Å), and N_2_ (3.6 Å) gases, as these are the primary components of pre and post‐combustion gas mixtures. Permeation experiments were carried out using a home‐made Wicke‐Kallenbach setup^[44]^ (Figure S4) at 303 K under constant transmembrane pressure. Across all membranes (both isotropic and anisotropic), pressure dependent N_2_ permeation at 303 K showed unaffected permeance (Figure S5), confirming absence of microscopic pinholes in the membranes.[^43^, ^45^] This finding correlates well with observations from cross‐section SEM images. For the neat ZIF‐8 membrane (~0.35 μm thickness), CO_2_, N_2_ and H_2_ permeance values were determined to be 9.5(±2)×10^−8^, 4.35(±1.5)×10^−8^ and 53.2(±12)×10^−8^ mol m^−2^ s^−1^ Pa^−1^ (averaged over ~2‐3 bar transmembrane pressure), respectively (Table S2). The permeance trend aligns with the kinetic diameter order, affirming a molecular sieving effect. For a thicker ZIF‐8 membrane (~0.65 μm; Figure S6) also we have observed similar selectivity, but lower permeance (Table S2). A comparison to the earlier reported neat ZIF‐8 membrane^[46]^ (using a LBL approach) indicates ~20 fold enhancement in CO_2_ permeance, while retaining the CO_2_/N_2_ ideal selectivity of ~2.2. The H_2_ permeance also enhanced by 22 fold compared to the earlier reported neat membrane ZIF‐8 membrane. The improved gas permeation with retention of the selectivity factor in the present case can be attributed to a high crystallinity, monolithic nature of the membrane. Also, see Table S2 for permeance values reported for comparable thickness ZIF‐8 membranes. Similarly, gas permeation experiments were also carried out for the UiO‐66‐NH_2_ membrane (~1.3 μm thickness). CO_2_, N_2_ and H_2_ permeance were found to be substantially high; 500.4(±27)×10^−8^, 625.5(±19)×10^−8^ and 2031(±53)×10^−8^ mol m^−2^ s^−1^ Pa^−1^ (averaged over ~1‐2 bar transmembrane pressure), respectively (Table S2). Higher permeance for N_2_ compared to CO_2_ indicates Knudsen type gas diffusion (calculated Knudsen selectivity N_2_/CO_2_=1.25; experimental idea selectivity N_2_/CO_2_=1.24). This can be attributed to the presence of substantially large void space in the UiO‐66‐NH_2_ due to missing linker or node defects (see Table S2).[^42^, ^47^] After confirming the gas permeation characteristics of the individual MOF membranes, we have compared the three different anisotropic membranes AAO‐UiO/ZIF 1–3. We have observed a constant decrease of gas permeance (averaged at 2–3 bar pressure) with increasing thickness of ZIF‐8 MOF, evident from the molecular sieving performance of the neat ZIF‐8 membrane (Figure 3a). However, the ideal CO_2_/N_2_ selectivity constantly increased from 1.45 to 2.8, surpassing the performance of neat ZIF‐8 membrane (Figure 3b). The lower selectivity for the thin layer ZIF‐8 membrane (UiO/ZIF 1) is possibly due to inhomogeneous growth of the top ZIF‐8 layer, while increasing thickness of ZIF‐8 converts the anisotropic membrane in a monolithic sieving layer. This observation correlates well with the cross‐section SEM images of the anisotropic membranes (Figure 2b–d). Next, we have compared the mixed‐gas separation performances of the neat sieving ZIF‐8 and AAO‐UiO/ZIF 3 membranes at 2 bar pressure, 303 K. The observed CO_2_/N_2_ selectivities are ~2 and ~2.9, and the corresponding CO_2_ permeance values are 4.5×10^−8^ and 1.4×10^−8^ mol m^−2^ s^−1^ Pa^−1^, respectively (Figure 3b). A comparison of the neat sieving ZIF‐8 and AAO‐UiO/ZIF 3 membranes confirms ~45 % enhanced selectivity (Figure 3b). We attribute this enhancement in permselectivity to the anisotropic pore distribution and the unique interface structure.

### Interface Effect on Gas Permeability

The interface between UiO‐66‐NH_2_ and ZIF‐8 consists of pores with different sizes from each MOF. Achieving precise alignment of these pores, as depicted in Scheme 1, requires specific chemical interactions to stabilize their orientation. However, due to lattice mismatch, achieving perfect alignment is challenging. Consequently, the interface exhibits misaligned pore windows from the individual MOFs, resulting in narrowed pores. We hypothesize that the creation of smaller interfacial pores contributes to enhanced size selectivity, as observed in the permselectivity of membranes, where AAO‐UiO/ZIF 3 shows greater CO_2_ selectivity than ZIF‐8.

**Figure 4:**
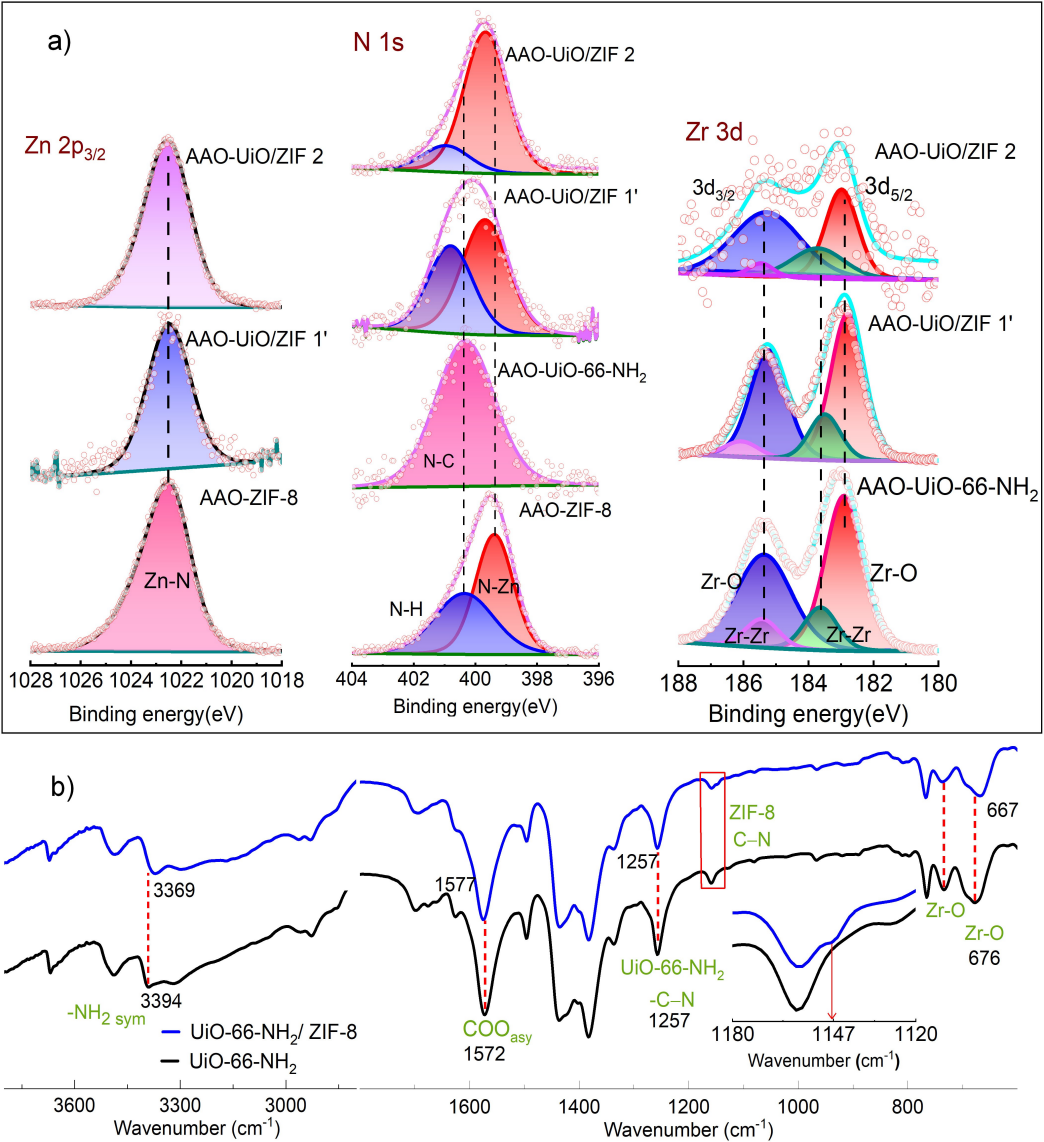
a) The XPS spectra of AAO‐ZIF‐8, AAO‐UiO‐66‐NH_2_, AAO‐UiO/ZIF 1’ and AAO‐UiO/ZIF 2 membranes for Zn, Zr, and N, solid lines are fittings. b) IRRA spectra of UiO‐66‐NH_2_ and UiO‐66‐NH_2_/ZIF‐8 thin film. Inset: enlarged view of C−N stretching vibration. Dotted lines are guide to the eyes.

Elucidating the creation of these smaller interfacial pores, formed by misaligned MOF pores, presents significant challenges, as evidenced in previous studies on MOF‐MOF heterostructures.[^31^, ^33^, ^37^] Nonetheless, it is evident that to form these constricted pores, ZIF‐8 and UiO‐66‐NH_2_ must undergo chemical crosslinking. To gain insight into the crosslinked functional groups of these MOFs, we characterized the thin films using XPS and IRRA (infrared reflection absorption) spectroscopy (Figure 4, S7). Note that these spectroscopic methods do not exclusively provide information about the interface. To enhance interface signal, we have grown thin layers of ZIF‐8 on top of UiO‐66‐NH_2_ MOF.

We anticipated that for strong interfacial adhesion of ZIF‐8 and UiO‐66‐NH_2_, Zn^2+^ ‐ bdc‐NH_2_ and Zr^4+^ – 2‐meIM interactions are crucial. Otherwise, MOF‐MOF interface can have intercrystalline voids. Comparison of Zn2p_3/2_ electron binding energy (1021 eV) confirmed that Zn coordination environment is similar for neat ZIF‐8 and a thin layer (10 LBL cycles) of ZIF‐8 on top of UiO‐66‐NH_2_ (AAO‐UiO/ZIF 1′) (Figure 4a). However, for N of 2‐meIM we have observed distinct changes. Compared to neat ZIF‐8, non‐coordinated N of 2‐meIm^[48]^ shifted to higher binding energy (400.45 to 400.85 eV) for the AAO‐UiO/ZIF 1′. For AAO‐UiO/ZIF 2 this binding energy shifted further to 400.95 eV. Also for Zn‐coordinated N similar shift to higher binding energy is observed, confirming that 2‐meIm also interacts with Zr^4+^ center of bottom layer.^[49]^ Note that −NH_2_ of UiO‐66‐NH_2_ also does contribute to these changes, and for further confirmation IRRAS is carried out (*vide infra*). The Zr3d_3/2_ and Zr3d_5/2_ binding energies decrease (0.1–0.2 eV) after thin layer of ZIF‐8 growth, indicating changes in the Zr^4+^ coordination of UiO‐66‐NH_2_ layer. Next, we have analyzed the IRRAS of neat UiO‐66‐NH_2_ and the thin layer of ZIF‐8 on top of UiO‐66‐NH_2_ (grown on functionalized Au surface, Figure 4b). Presence of ZIF‐8 top layer is evident from the new Zn−N vibration appeared at 1147 cm^−1^.^[50]^ From Figure 4b it is evident that symmetric −NH_2_ vibration^[51]^ at 3394 cm^−1^ shifted to 3369 cm^−1^, confirming that NH_2_ interacts with Zn^2+^. This was not obvious from the XPS, due to multiple overlapped peaks. Further the Zr−O stretching vibration^[52]^ also shifted from 676 to 667 cm^−1^, confirming changes in the Zr^4+^ coordination environment at the interface. Correlating the observations from XPS and IRRAS, we conclude a chemical crosslinking of ZIF‐8 and UiO‐66‐NH_2_, i. e. small and large pore, via Zn^2+^ ‐ bdc‐NH_2_ and Zr^4+^ – 2‐meIM interactions. This chemical crosslinking allows a monolith formation, as can be seen from the cross‐section SEM image of AAO‐UiO/ZIF 3 (Figure 2d). The observed high permselectivity, pinhole free membrane structure and chemical crosslinking between the two very different MOF‐layers substantiate a gas diffusion pathway akin to that depicted as path B, in Scheme 1. The earlier report on similar MOF‐MOF interface, however, did not provide any evidence of interface crosslinking. Our observations conclude that indeed interface chemical structure (smaller interfacial pores or dangling chemical functionalities) boost CO_2_/N_2_ selectivity.

Further, we have compared the CO_2_/CH_4_ (CH_4_ kinetic diameter~3.8 Å) ideal gas selectivity at 303 K. In the presence of interface void and pinhole, a CH_4_ permeance should be higher than CO_2_ (according to Knudsen diffusion). However we have observed a CO_2_ selectivity of 2.1 over CH_4_ (permeance ~1.5×10^−8^ mol m^−2^ s^−1^ Pa^−1^) for the UiO/ZIF 3 membrane. This observation reinstates our hypothesis of pinhole free, chemically cross‐linked interface.

## Conclusions

In conclusion, we have successfully developed a de novo method to engineer an anisotropic dual‐pore membrane structure, facilitating rapid and selective gas diffusion. This involved crafting a heterolayer structure using two MOFs, ZIF‐8 and UiO‐66‐NH_2_, with differing pore sizes and topologies in a precise, stepwise manner. Our optimized synthesis technique enables the creation of a monolithic membrane with a chemically crosslinked interface. The resulting membrane exhibits enhanced CO_2_ permeability and CO_2_/N_2_ selectivity compared to individual MOFs, confirming the effectiveness of intercrystalline void‐free interfaces and large pore – small pore chemical crosslinking. This novel lattice mismatched MOF‐on‐MOF heterolayer membrane approach is a significant advancement towards designing new membrane structure. While analyzing the chemical structure of this unique MOF‐MOF interface presents challenges, advanced computational modeling can provide further insights. Our breakthrough in creating this lattice‐mismatched MOF interface, without any chemical modification, opens doors for further exploration of distinctive MOF interfaces in future research.

## Supporting Information Summary

The authors have cited additional references within the Supporting Information.[^30^, ^31^]

## Conflict of Interests

The authors declare no conflict of interest.

1

## Supporting information

As a service to our authors and readers, this journal provides supporting information supplied by the authors. Such materials are peer reviewed and may be re‐organized for online delivery, but are not copy‐edited or typeset. Technical support issues arising from supporting information (other than missing files) should be addressed to the authors.

Supporting Information

## Data Availability

The data that support the findings of this study are available from the corresponding author upon reasonable request.

## References

[1] Q. Qian , P. A. Asinger , M. J. Lee , G. Han , K. Mizrahi Rodriguez , S. Lin , F. M. Benedetti , A. X. Wu , W. S. Chi , Z. P. Smith , Chem. Rev. 2020, 120, 8161–8266. 32608973 10.1021/acs.chemrev.0c00119

[2] D. S. Sholl , R. P. Lively , Nature 2016, 532, 435–437. 27121824 10.1038/532435a

[3] A. Knebel , J. Caro , Nat. Nanotechnol. 2022, 17, 911–923. 35995854 10.1038/s41565-022-01168-3

[4] O. Smirnova , S. Hwang , R. Sajzew , L. Ge , A. Reupert , V. Nozari , S. Savani , C. Chmelik , M. R. Reithofer , L. Wondraczek , J. Kärger , A. Knebel , Nat. Mater. 2024, 23, 262–270. 38123813 10.1038/s41563-023-01738-3 PMC10837076

[5] S. Guo , J. Y. Yeo , F. M. Benedetti , D. Syar , T. M. Swager , Z. P. Smith , Angew. Chem. Int. Ed. 2024, 63, e202315611. 10.1002/anie.202315611 38084884

[6] A. Huang , J. Caro , Angew. Chem. Int. Ed. 2011, 50, 4979–4982. 10.1002/anie.201007861 21472930

[7] O. Smirnova, S. Ojha, A. De, A. Schneemann, F. Haase, A. Knebel, *Adv. Funct. Mater*. n/a, 2306202.

[8] H. Furukawa , N. Ko , Y. B. Go , N. Aratani , S. B. Choi , E. Choi , A. Ö. Yazaydin , R. Q. Snurr , M. O'Keeffe , J. Kim , O. M. Yaghi , Science 2010, 329, 424–428. 20595583 10.1126/science.1192160

[9] T. Xu , P. Zhang , F. Cui , J. Li , L. Kan , B. Tang , X. Zou , Y. Liu , G. Zhu , Adv. Mater. 2023, 35, 2204553. 10.1002/adma.202204553 36573630

[10] S. Horike , S. Shimomura , S. Kitagawa , Nat. Chem. 2009, 1, 695–704. 21124356 10.1038/nchem.444

[11] Y. Feng , Z. Wang , W. Fan , Z. Kang , S. Feng , L. Fan , S. Hu , D. Sun , J. Mater. Chem. A 2020, 8, 13132–13141.

[12] S. Cong , Y. Zhou , C. Luo , C. Wang , J. Wang , Z. Wang , X. Liu , Angew. Chem. Int. Ed. 2024, 63, e202319894. 10.1002/anie.202319894 38265268

[13] C.-K. Chang , T.-R. Ko , T.-Y. Lin , Y.-C. Lin , H. J. Yu , J. S. Lee , Y.-P. Li , H.-L. Wu , D.-Y. Kang , Commun. Chem. 2023, 6, 118. 37301865 10.1038/s42004-023-00917-2 PMC10257696

[14] S. Kundu , R. Haldar , Dalton Trans. 2023, 52, 15253–15276. 37603374 10.1039/d3dt01878d

[15] S. Roy , Z. Huang , A. Bhunia , A. Castner , A. K. Gupta , X. Zou , S. Ott , J. Am. Chem. Soc. 2019, 141, 15942–15950. 31508946 10.1021/jacs.9b07084 PMC6803166

[16] L. Ye , J. Liu , Y. Gao , C. Gong , M. Addicoat , T. Heine , C. Wöll , L. Sun , J. Mater. Chem. A 2016, 4, 15320–15326.

[17] A. Chandresh , X. Liu , C. Wöll , L. Heinke , Adv. Sci. 2021, 8, 2001884. 10.1002/advs.202001884 PMC8024988 33854871

[18] Y. Wu , Y. Li , J. Gao , Q. Zhang , SusMat 2021, 1, 66–87.

[19] W. Li , S. Xue , S. Watzele , S. Hou , J. Fichtner , A. L. Semrau , L. Zhou , A. Welle , A. S. Bandarenka , R. A. Fischer , Angew. Chem. Int. Ed. 2020, 59, 5837–5843. 10.1002/anie.201916507 PMC7154533 31912955

[20] Q. Xia , Z. Li , C. Tan , Y. Liu , W. Gong , Y. Cui , J. Am. Chem. Soc. 2017, 139, 8259–8266. 28537723 10.1021/jacs.7b03113

[21] H. Deng , C. J. Doonan , H. Furukawa , R. B. Ferreira , J. Towne , C. B. Knobler , B. Wang , O. M. Yaghi , Science 2010, 327, 846–850. 20150497 10.1126/science.1181761

[22] M. Kalaj , S. M. Cohen , ACS Cent. Sci. 2020, 6, 1046–1057. 32724840 10.1021/acscentsci.0c00690 PMC7379093

[23] Z. Fang , B. Bueken , D. E. De Vos , R. A. Fischer , Angew. Chem. Int. Ed. 2015, 54, 7234–7254. 10.1002/anie.201411540 PMC4510710 26036179

[24] S. Dai , C. Simms , I. Dovgaliuk , G. Patriarche , A. Tissot , T. N. Parac-Vogt , C. Serre , Chem. Mater. 2021, 33, 7057–7066.

[25] S. Li , W. Han , Q.-F. An , K.-T. Yong , M.-J. Yin , Adv. Funct. Mater. 2023, 33, 2303447.

[26] W. Fan , Y. Ying , S. B. Peh , H. Yuan , Z. Yang , Y. D. Yuan , D. Shi , X. Yu , C. Kang , D. Zhao , J. Am. Chem. Soc. 2021, 143, 17716–17723. 34608802 10.1021/jacs.1c08404

[27] K. Hirai , S. Furukawa , M. Kondo , H. Uehara , O. Sakata , S. Kitagawa , Angew. Chem. Int. Ed. 2011, 50, 8057–8061. 10.1002/anie.201101924 21761521

[28] T. Li , J. E. Sullivan , N. L. Rosi , J. Am. Chem. Soc. 2013, 135, 9984–9987. 23795996 10.1021/ja403008j

[29] M.-X. Wu , Y. Wang , G. Zhou , X. Liu , ACS Appl. Mater. Interfaces 2020, 12, 54285–54305. 33231416 10.1021/acsami.0c16428

[30] G. Lee , S. Lee , S. Oh , D. Kim , M. Oh , J. Am. Chem. Soc. 2020, 142, 3042–3049. 31968935 10.1021/jacs.9b12193

[31] Z. Song , F. Qiu , E. W. Zaia , Z. Wang , M. Kunz , J. Guo , M. Brady , B. Mi , J. J. Urban , Nano Lett. 2017, 17, 6752–6758. 29072837 10.1021/acs.nanolett.7b02910

[32] H. Li , S. Zhuang , B. Zhao , Y. Yu , Y. Liu , Sep. Purif. Technol. 2023, 305, 122504.

[33] K. Ikigaki , K. Okada , Y. Tokudome , T. Toyao , P. Falcaro , C. J. Doonan , M. Takahashi , Angew. Chem. Int. Ed. 2019, 58, 6886–6890. 10.1002/anie.201901707 30924218

[34] R. Banerjee , A. Phan , B. Wang , C. Knobler , H. Furukawa , M. O'Keeffe , O. M. Yaghi , Science 2008, 319, 939–943. 18276887 10.1126/science.1152516

[35] J. H. Cavka , S. Jakobsen , U. Olsbye , N. Guillou , C. Lamberti , S. Bordiga , K. P. Lillerud , J. Am. Chem. Soc. 2008, 130, 13850–13851. 18817383 10.1021/ja8057953

[36] A. L. Semrau , R. A. Fischer , Chem. A Eur. J. 2021, 27, 8509–8516. 10.1002/chem.202005416 PMC8251636 33830544

[37] Z. Wang , J. Liu , B. Lukose , Z. Gu , P. G. Weidler , H. Gliemann , T. Heine , C. Wöll , Nano Lett. 2014, 14, 1526–1529. 24512342 10.1021/nl404767k

[38] M. Oldenburg , A. Turshatov , D. Busko , S. Wollgarten , M. Adams , N. Baroni , A. Welle , E. Redel , C. Wöll , B. S. Richards , I. A. Howard , Adv. Mater. 2016, 28, 8477–8482. 27500466 10.1002/adma.201601718

[39] R. Haldar , C. Wöll , Nano Res. 2021, 14, 355–368.

[40] S. Choi , T. Kim , H. Ji , H. J. Lee , M. Oh , J. Am. Chem. Soc. 2016, 138, 14434–14440. 27728969 10.1021/jacs.6b08821

[41] D. H. Hong , H. S. Shim , J. Ha , H. R. Moon , Bull. Korean Chem. Soc. 2021, 42, 956–969.

[42] P. Sindhu, S. Saha, U. Bhoi, N. Ballav, *Adv. Funct. Mater. n/a*, 2312515.

[43] E. P. Valadez Sánchez , H. Gliemann , K. Haas-Santo , C. Wöll , R. Dittmeyer , Chem. Ing. Tech. 2016, 88, 1798–1805.

[44] H. Bux , F. Liang , Y. Li , J. Cravillon , M. Wiebcke , J. Caro , J. Am. Chem. Soc. 2009, 131, 16000–16001. 19842668 10.1021/ja907359t

[45] M. C. McCarthy , V. Varela-Guerrero , G. V. Barnett , H.-K. Jeong , Langmuir 2010, 26, 14636–14641. 20731336 10.1021/la102409e

[46] O. Shekhah , R. Swaidan , Y. Belmabkhout , M. du Plessis , T. Jacobs , L. J. Barbour , I. Pinnau , M. Eddaoudi , Chem. Commun. 2014, 50, 2089–2092. 10.1039/c3cc47495j 24448609

[47] H. Guo , J. Liu , Y. Li , J. Caro , A. Huang , Microporous Mesoporous Mater. 2021, 313, 110823.

[48] Q. Liu , Y. Miao , L. F. Villalobos , S. Li , H.-Y. Chi , C. Chen , M. T. Vahdat , S. Song , D. J. Babu , J. Hao , Y. Han , M. Tsapatsis , K. V. Agrawal , Nat. Mater. 2023, 22, 1387–1393. 37735526 10.1038/s41563-023-01669-z PMC10627807

[49] X. Fang , S. Wu , Y. Wu , W. Yang , Y. Li , J. He , P. Hong , M. Nie , C. Xie , Z. Wu , K. Zhang , L. Kong , J. Liu , Appl. Surf. Sci. 2020, 518, 146226.

[50] D. Huang , Q. Xin , Y. Ni , Y. Shuai , S. Wang , Y. Li , H. Ye , L. Lin , X. Ding , Y. Zhang , RSC Adv. 2018, 8, 6099–6109. 35539600 10.1039/c7ra09794h PMC9078250

[51] D. T. Lee , J. Zhao , C. J. Oldham , G. W. Peterson , G. N. Parsons , ACS Appl. Mater. Interfaces 2017, 9, 44847–44855. 29165990 10.1021/acsami.7b15397

[52] P. C. Lemaire , D. T. Lee , J. Zhao , G. N. Parsons , ACS Appl. Mater. Interfaces 2017, 9, 22042–22054. 28598598 10.1021/acsami.7b05214

