# Supplementary material for: Anisotropic Porosity and Interface Synergy Enhanced Gas Permselectivity in Heterolayer Metal‐Organic Framework Membrane
Source: Chemistry. 2024 Nov 12;30(72):e202403607. doi: 10.1002/chem.202403607 (PMC11665486; doi:10.1002/chem.202403607)
Supplement: Supplementary file 1 — Supporting Information [file CHEM-30-e202403607-s001.pdf]

Chemistry–A European Journal

Supporting Information

Anisotropic Porosity and Interface Synergy Enhanced Gas Permselectivity in Heterolayer Metal-Organic Framework Membrane

Susmita Kundu, Tanmoy Maity, Suvendu Panda, and Ritesh Haldar*

Supporting Information

Anisotropic porosity and interface synergy enhanced gas permselectivity in heterolayer metal-organic framework membrane

Susmita Kundu, Tanmoy Maity, Suvendu Panda, and Ritesh Haldar*

Tata Institute of Fundamental Research Hyderabad, Gopanpally, Hyderabad 500046, Telangana, India

Materials

Zirconium chloride, zinc nitrate hexahydrate, benzoic acid, 1,4-benzenedicarboxylic acid, 2-aminobenzene-1,4-dicarboxylic acid and 2-methylimidazole were purchased from Sigma-Aldrich. N,N-dimethylformamide (DMF), methanol, isopropanol (HPLC grade), acetic acid and hydrochloric acid were purchased from Thermo Fisher Scientific. All chemicals were used as received without further purification. The porous anodic aluminum oxide (AAO) substrates (isotropic) with ~200 nm pore diameter and ~50 µm thickness was supplied by SPI Supplies.

Methods:

UiO-66-NH_2 seeding solution: UiO-66-NH_2 powder was prepared using the method described by Uemiya.[1] A mixture of $ZrCl_4$, 2-aminoterephthalic acid, water, acetic acid, and DMF with a molar ratio of 1:1:1:500:750 was heated in a Teflon lined autoclave at 120 ºC for 24 hours. After cooling to room temperature, the powder was collected by centrifugation and washed several times with DMF and ethanol. The as synthesized powder was then suspended in ethanol (~100 mg/ml) to prepare the seeding solution.

UiO-66-NH_2 synthesis solution: $ZrCl_4$ (420 mg, 3.6 mmol) was dissolved in 4.5 ml DMF at 150 °C and 2-aminoterephthalic acid (305 mg, 1.71 mmol) with benzoic acid (4.4 g, 36 mmol) were dissolved in 10 ml DMF separately at 150 °C. After cooling them to room temperature, both solutions were mixed to make the synthesis solution.

Synthesis of UiO-66-NH_2 membranes: Gold-coated silicon wafers were dipped in an ethanolic solution (20 mM) of 11-mercapto-1-undecanol (MUD) for 24 hours to obtain –OH functionalized surface. These substrates were then thoroughly washed with absolute ethanol (99.99%), dried and used for membranes synthesis. SiO_2/Si wafers were cleaned by isopropanol and then by UV-ozone cleaner, to remove organic impurities and to create free –OH groups on the surface. The AAO support was subsequently cleaned with deionized water and isopropanol respectively. The cleaned AAO was dipped in 6% aqueous HCl solution for 6 hours. The acid-treated substrate was washed thoroughly with ultrapure water and ethanol. After UV-ozone cleaning for 20 minutes, the Si wafer-supported AAO substrate (back side of the AAO was supported on a Si wafer) was dipped inside the homogeneous seeding solution (preparation of seeding solution discussed above). After removing from the seeding solution the seeded-AAO was heated at 80 ºC in a preheated oven for 2 hours. The seeded-AAO support was then dipped into the synthesis solution and heated at 120 °C for 24 hours to make the membrane. After the reaction, the solution was cooled to room temperature, and the AAO-supported membrane was washed thoroughly with DMF and ethanol. The membrane was kept in fresh ethanol for 48 hours (solvent exchange) and heated inside a vacuum oven for 12 hours to activate the membrane pores. Additional activation of the UiO-66-NH_2 membrane was carried out within with the flow cell at 60 °C under continuous N_2 flow.

Synthesis of ZIF-8 membrane: The ZIF-8 membrane was prepared on AAO following a reported method described by Dittmeyer.[2] The -OH functionalized AAO substrate was subsequently immersed into the following metal, linker, and rinsing solution with the help of a dipping robot: (a) 10 mM zinc nitrate hexahydrate solution for 300 seconds, (b) methanol for 100 seconds, (c) 20 mM 2-methylimidazole for 300 seconds, and (d) methanol for 100 seconds. After the completion of 200 cycles, the membrane was washed thoroughly with methanol and activated under N_2 flow at 60 °C inside the flow cell.

Synthesis of AAO-UiO/ZIF 1-3 membranes: The UiO-66-NH_2 membranes were prepared by the method as described above. After the solvent exchange process with ethanol for 48 hours, ZIF-8 layer was grown on top of the UiO-66-NH_2 membranes for 150, 200, and 250 cycles (ZIF-8 thickness: 0.4 ±0.2 μm, 0.5 ±0.1 μm and 1 ±0.2 μm, respectively) using the layer-by-layer LPE method as described above (AAO-UiO/ZIF 1-3, respectively). The

anisotropic membranes were first activated inside the vacuum oven at 130 °C for 12 hours and then inside the flow cell at 60 °C under N_2 flow.

Gas permeance experiment:

Pristine and bilayer membranes were mounted inside a homemade setup as shown in Figure S5. During measurements, single gases (CO_2, N_2, CH_4, and H_2) and a mixture of gases (50:50 CO_2/N_2) were used as feed. For the permeance experiment, the permeate gas flow rates are measured using a bubble flow meter. The composition of the permeate gas mixture in the case of mixed gas permeance is measured through gas chromatography. On the permeate side, Argon was used as a sweep gas. Gas permeance and permeability are calculated using the following equations.

$$Permeance = \frac{n}{A * \Delta p}$$

$$Permeability = Permeance * L$$

Where, n is the amount of gas passing through the membrane, A is the effective area of the membrane, ΔP is transmembrane pressure difference, and L is the thickness of the membrane. The Knudsen selectivity for the gases P and Q can be calculated by the following equation[3]

$$\alpha_{ideal}\left(\frac{p}{Q}\right) = \frac{\sqrt{M(P)}}{\sqrt{M(Q)}}$$

Characterization techniques

Powder X-ray diffractometer (XRD) patterns of thin films were recorded on a Rigaku XDS 2000 diffractometer using nickel-filtered Cu Kα radiation (λ = 1.5418 Å) ranging from 5 to 20 ° at room temperature (voltage 40 kV, current 200 mA). Out-of-plane PXRD was recorded in $2\theta/\theta$ (step size 0.01, scan rate 0.4 °/s), in-plane in $2\theta/\varphi$ geometry with grazing incident angle (ω) at 0.5 ° and step size of 0.12 with scan rate 0.1 °/s.

Surface morphology of samples were characterized using field emission scanning electron microscopy (FESEM), JEOL JSM-7200F instrument with a cold emission gun operating at 30 kV. Energy-Dispersive X-ray spectroscopy (EDS) elemental analysis and mapping were also done on the FESEM.

IRRA (Infrared Reflection Absorption) spectrum (4000–600 cm^{-1}) was collected under vacuum using Bruker VERTEX 70v with 2 cm^{-1} resolution and with 128 scan rate.

X-ray photoelectron spectroscopy: Elemental (Zn, Zr and Al) depth profiling (etching rate 5.75 nm/min) of the thin film was performed using x-ray photoelectron spectrometer (PHI versaProbe III) within an ultrahigh vacuum (1×10^{-9} bar) environment. This instrument was equipped with an Al $K\alpha$ x-ray source and a monochromator. For ion sputtering, Ar gas was used at 2 kV, over a 2×2 mm^2 area at an angle of 45° to the surface normal. The atomic composition was determined based on photoelectron peak areas and the relative sensitivity factors provided in PHI's MultiPak processing software.

Gas chromatography: The gas outlet of the flow cell was directly connected to the inlet of the gas chromatograph instrument (Thermo Trace GC-1110), equipped with a molecular sieve column with He as the carrier gas (flow rate: 20 mL min^{-1}). CO_2 and N_2 gases were detected by a thermal conductivity detector (TCD).

Table S1: Cell parameters and space group of UiO-66-NH_2 and ZIF-8 MOF.[4-5]

MOF	Unit cell parameter(Å)			Cell angle(°)			Cell volume (Å^3)	Space group	CCDC
	a	b	c	α	β	γ			
UiO-66-NH_2	20.7988	20.7988	20.7988	90	90	90	8997.4	*Fm*-3*m*	1405751
ZIF-8	16.8815	16.8815	16.8815	90	90	90	4810.97	*I*-43*m*	1429243

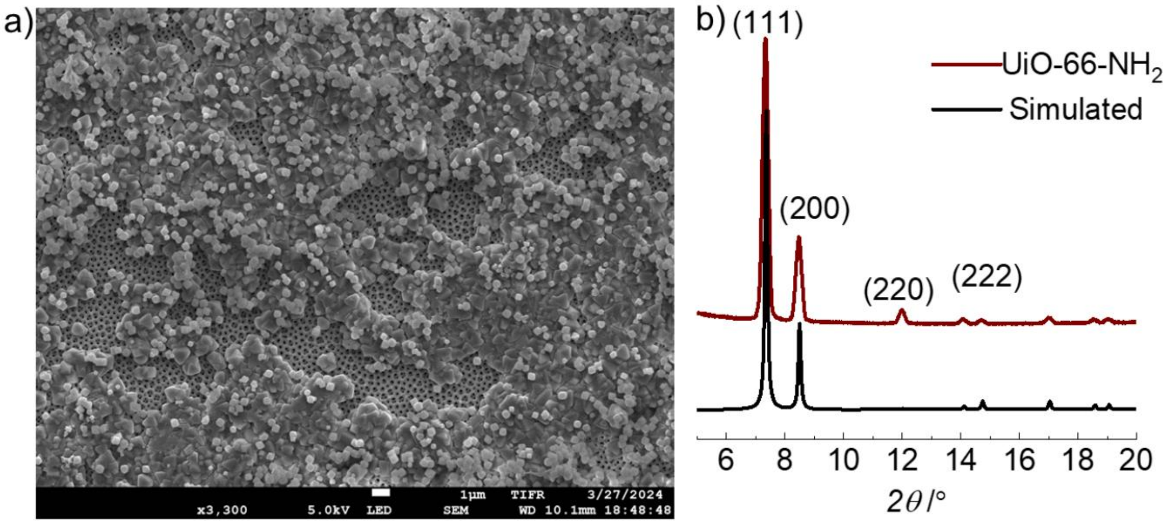

Figure S1: a) Inhomogeneous deposition of UiO-66-NH_2 materials on AAO using solvothermal method without seeding. AAO pores are visible here. b) XRD of the inhomogeneously deposited UiO-66-NH_2 on AAO.

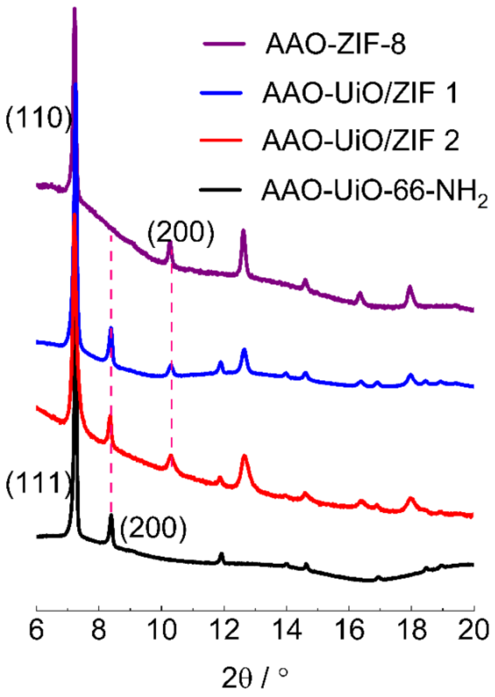

Figure S2: XRD patterns of AAO-UiO/ZIF 1 and AAO-UiO/ZIF 2 membranes along with the pristine UiO-66-NH_2 and ZIF-8 membranes.

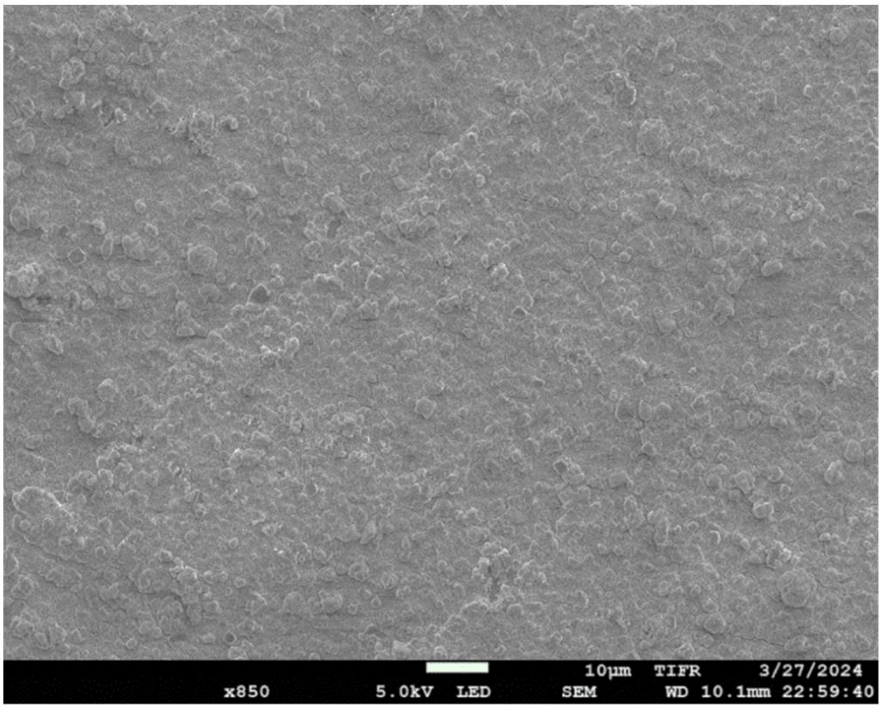

Figure S3: Large area SEM image of AAO-UiO/ZIF 3 membrane.

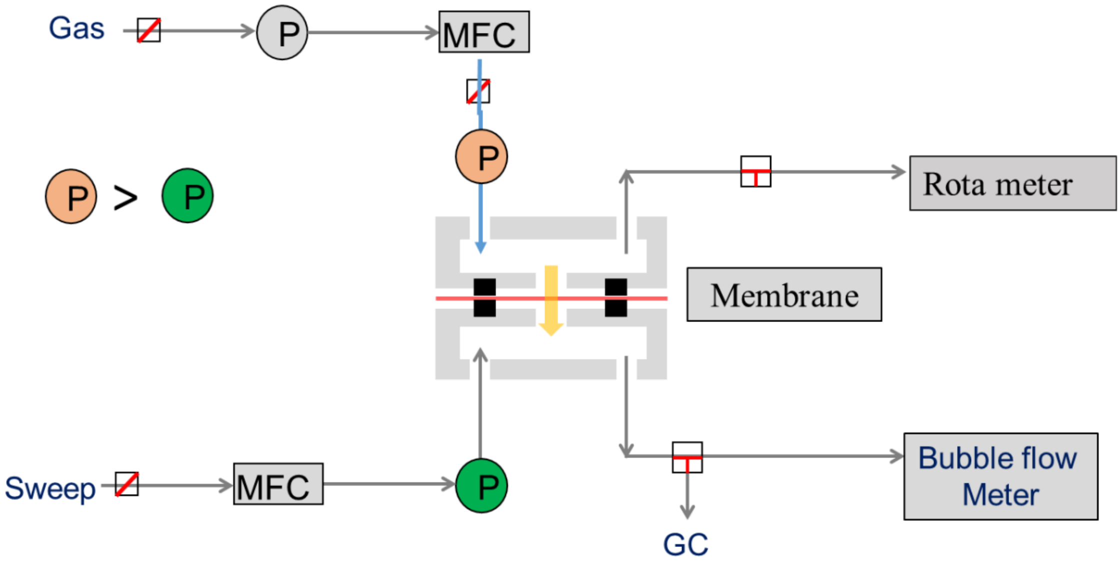

Figure S4: Wicke-Kallenbach setup for gas permeance experiment. MFC = mass flow meter.

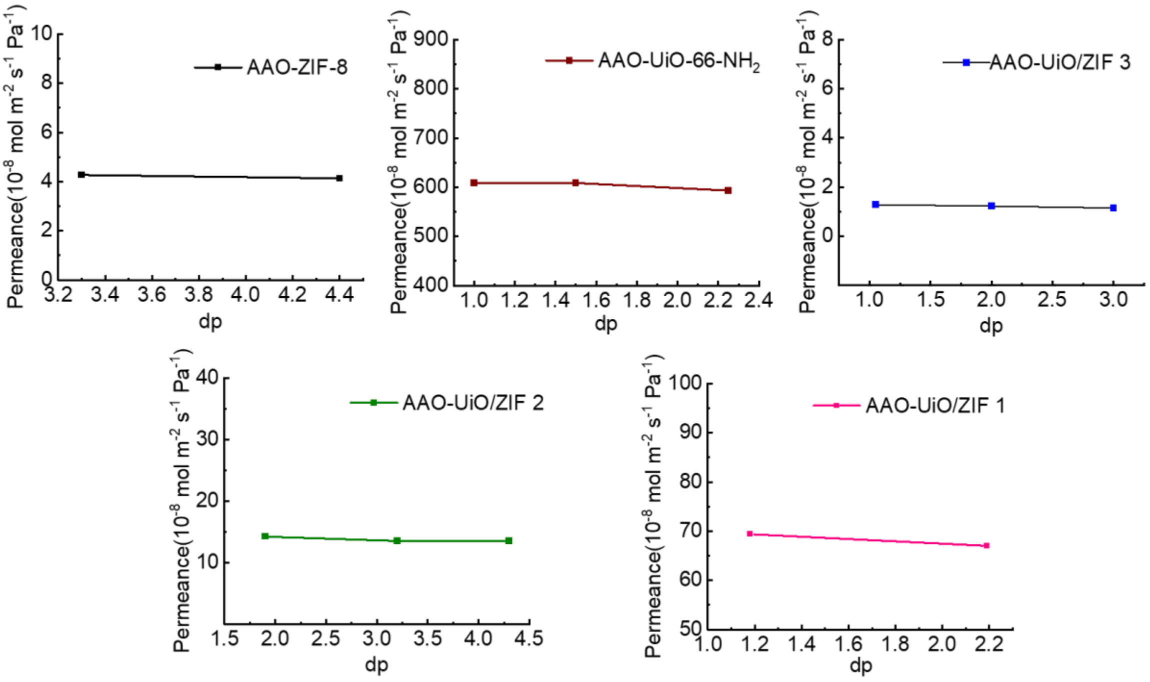

Figure S5: Pressure dependent N_2 permeance for all the membranes (AAO-ZIF-8, AAO-UiO-66-NH_2, AAO-UiO/ZIF 3, AAO-UiO/ZIF 2, and AAO-UiO/ZIF 1. dp = transmembrane pressure.

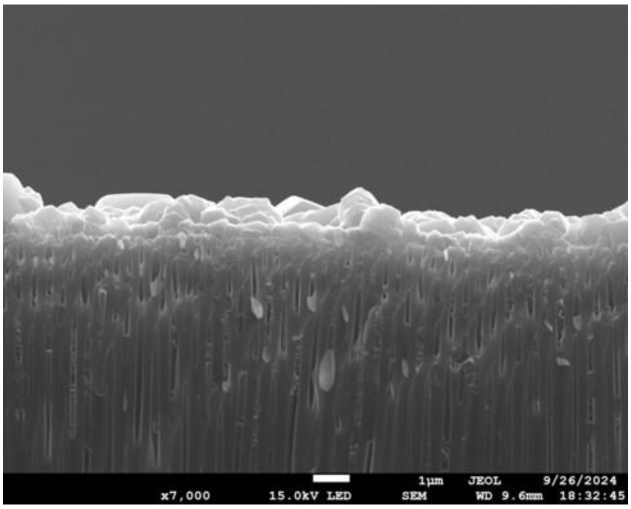

Figure S6: Cross-section SEM of thicker ZIF-8 membrane.

Table S2: CO_2 permeance and CO_2/N_2 selectivity for ZIF-8 and UiO-66-NH_2 membranes. This work is denoted with *.

MOF membrane	CO_2 permeance(10^{-8} mol m^{-2} s^{-1} Pa^{-1})	CO_2/N_2 selectivity	Thickness (µm)	Methodology
ZIF-8[6]	1.33	1.56	40	Microwave
ZIF-8[7]	113	2.13	0.5	Electrophoretic Nuclei Assembly
ZIF-8[8]	0.41	2.15	1.6	LPE
ZIF-8[9]	4.45	2.9	20	Solvothermal, 120°C
ZIF-8[10]	2.10	1.70	20	Solvothermal, 85°C
ZIF-8[11]	302	1.75	30	Solvothermal, 85°C
ZIF-8[12]	3.3	2.3	20	Solvothermal, 75°C
ZIF-8[13]	3.4	1.62	6	Solvothermal, 100°C
ZIF-8[14]	1.19	2.97	0.002	RT
ZIF-8[15]	50	3.2	0.8	Electrophoretic nuclei assembly
UiO-66-NH_2[16]	27.3	18.5	1	Solvothermal (Desalination)
UiO-66-NH_2[17]	1.23	0.46 (N_2> CO_2)	1.2	Microwave
UiO-66-NH_2[18]	2.12	0.6 (N_2> CO_2)	3	Solvothermal
UiO-66[3]	10	0.6 (N_2>CO_2)	5	Solvothermal
UiO-66[19]	2.6	2.66	15	Solvothermal
UiO-66[20]	3.8	36.5	1.8	Solvothermal
UiO-66[21]	40.7	35.6	2.2	Solvothermal
UiO-66[22]	-	37.8		RT
ZIF-8*	9.5	2.2	0.35	Layer-by-layer (LBL)
ZIF-8*	5.1	2.2	0.66	LBL
UiO-66-NH_2*	500	0.8	1.7	Solvothermal
UiO/ZIF 1 *	98	1.48	2	Solvothermal+LBL

UiO/ZIF 2*	25	1.86	1.85	Solvothermal+LBL
UiO/ZIF 3*	3.1	2.8	2.5	Solvothermal+LBL

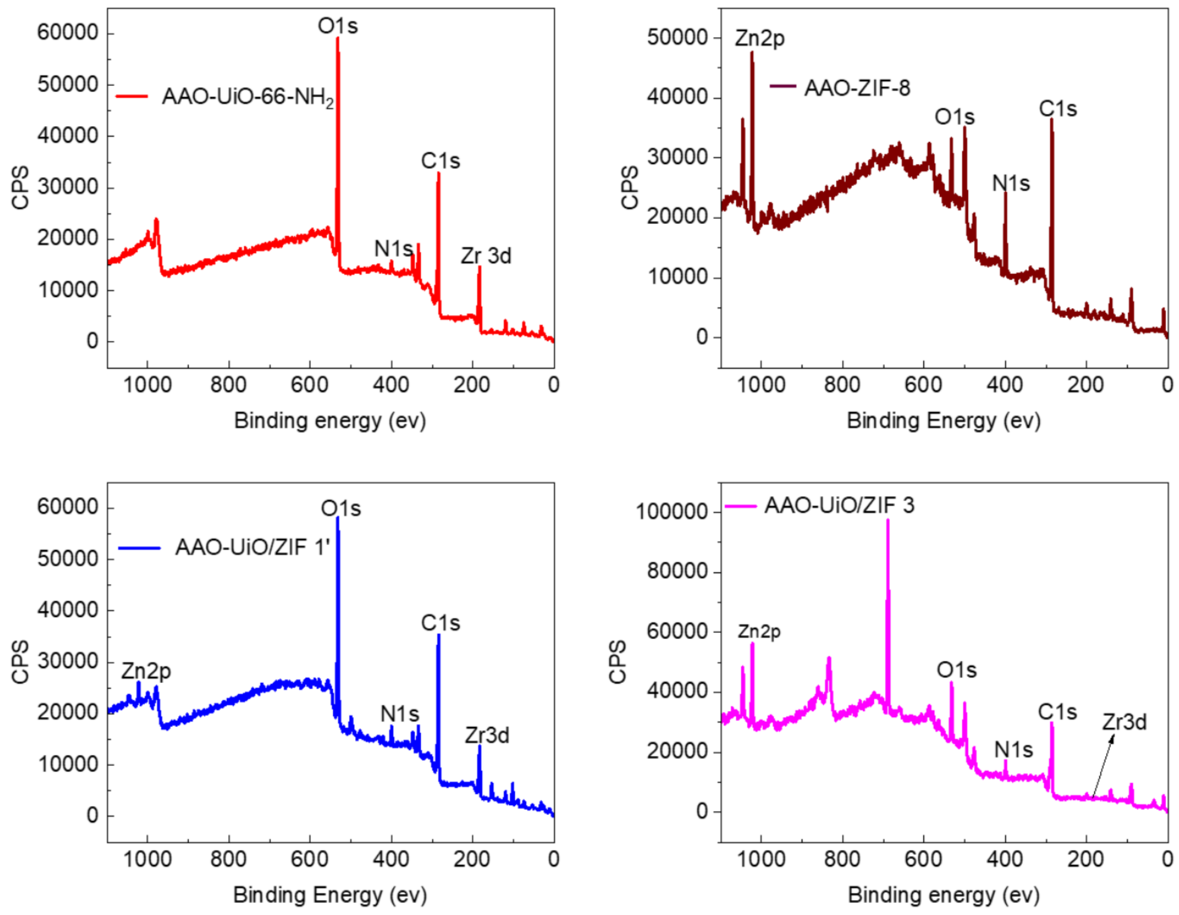

Figure S7: XPS spectra of AAO-UiO-66-NH_2, AAO-ZIF-8, AAO-UiO/ZIF 1’, and AAO-UiO/ZIF 3 membranes.

References

[1] M. Miyamoto, K. Hori, T. Goshima, N. Takaya, Y. Oumi, S. Uemiya, *European Journal of Inorganic Chemistry* **2017**, *2017*, 2094-2099.

[2] E. P. Valadez Sánchez, H. Gliemann, K. Haas-Santo, C. Wöll, R. Dittmeyer, *Chemie Ingenieur Technik* **2016**, *88*, 1798-1805.

[3] S. Friebe, B. Geppert, F. Steinbach, J. Caro, *ACS Applied Materials & Interfaces* **2017**, *9*, 12878-12885.

[4] R. Banerjee, A. Phan, B. Wang, C. Knobler, H. Furukawa, M. O'Keeffe, O. M. Yaghi, *Science* **2008**, *319*, 939-943.

[5] A. L. Semrau, R. A. Fischer, *Chemistry – A European Journal* **2021**, *27*, 8509-8516.

[6] H. Bux, F. Liang, Y. Li, J. Cravillon, M. Wiebcke, J. Caro, *Journal of the American Chemical Society* **2009**, *131*, 16000-16001.

[7] G. He, M. Dakhchoune, J. Zhao, S. Huang, K. V. Agrawal, *Advanced Functional Materials* **2018**, *28*, 1707427.
[8] O. Shekhah, R. Swaidan, Y. Belmabkhout, M. du Plessis, T. Jacobs, L. J. Barbour, I. Pinnau, M. Eddaoudi, *Chemical Communications* **2014**, *50*, 2089-2092.
[9] M. C. McCarthy, V. Varela-Guerrero, G. V. Barnett, H.-K. Jeong, *Langmuir* **2010**, *26*, 14636-14641.
[10] Q. Liu, N. Wang, J. Caro, A. Huang, *Journal of the American Chemical Society* **2013**, *135*, 17679-17682.
[11] A. Huang, Q. Liu, N. Wang, J. Caro, *Journal of Materials Chemistry A* **2014**, *2*, 8246-8251.
[12] Y. Liu, N. Wang, J. H. Pan, F. Steinbach, J. Caro, *Journal of the American Chemical Society* **2014**, *136*, 14353-14356.
[13] X. Zhang, Y. Liu, S. Li, L. Kong, H. Liu, Y. Li, W. Han, K. L. Yeung, W. Zhu, W. Yang, J. Qiu, *Chemistry of Materials* **2014**, *26*, 1975-1981.
[14] Q. Liu, Y. Miao, L. F. Villalobos, S. Li, H.-Y. Chi, C. Chen, M. T. Vahdat, S. Song, D. J. Babu, J. Hao, Y. Han, M. Tsapatsis, K. V. Agrawal, *Nature Materials* **2023**, *22*, 1387-1393.
[15] J. Hao, D. J. Babu, Q. Liu, P. A. Schouwink, M. Asgari, W. L. Queen, K. V. Agrawal, *Chemistry of Materials* **2021**, *33*, 4035-4044.
[16] L. Wan, C. Zhou, K. Xu, B. Feng, A. Huang, *Microporous and Mesoporous Materials* **2017**, *252*, 207-213.
[17] Y. Sun, C. Song, X. Guo, Y. Liu, *ACS Applied Materials & Interfaces* **2020**, *12*, 4494-4500.
[18] H. Guo, J. Liu, Y. Li, J. Caro, A. Huang, *Microporous and Mesoporous Materials* **2021**, *313*, 110823.
[19] B. Shan, J. B. James, M. R. Armstrong, E. C. Close, P. A. Letham, K. Nikkhah, Y. S. Lin, B. Mu, *The Journal of Physical Chemistry C* **2018**, *122*, 2200-2206.
[20] J. Yan, Y. Sun, T. Ji, Y. Liu, N. Zhang, B. Sun, S. Meng, B. H. Yin, M. Wu, H. Hu, Y. Liu, *Industrial & Engineering Chemistry Research* **2023**, *62*, 5973-5983.
[21] J. Yan, Y. Sun, T. Ji, L. Liu, M. Zhang, Y. Liu, *Journal of Membrane Science* **2021**, *635*, 119515.
[22] J. Yan, Y. Sun, T. Ji, C. Zhang, L. Liu, Y. Liu, *Journal of Membrane Science* **2022**, *653*, 120496.
